# The effect of rigorous study design in the research of autologous bone marrow-derived mononuclear cell transfer in patients with acute myocardial infarction

**DOI:** 10.1186/scrt233

**Published:** 2013-07-12

**Authors:** Hyunsuk Jeong, Hyeon Woo Yim, Youngseung Cho, Hun Jun Park, Sona Jeong, Hyun-bin Kim, Wonhee Hong, Heejung Kim

**Affiliations:** 1 Department of Preventive Medicine, The Catholic University of Korea, College of Medicine, 505 Banpo-dong, Seocho-gu, Seoul, Republic of Korea; 2 Occupational and Environmental Medicine, Seoul, Republic of Korea; 3 Division of Cardiovascular Medicine, Seoul St. Mary’s Hospital, Seoul, Republic of Korea; 4 Medical Library, Yeouido St. Mary’s Hospital, Seoul, Republic of Korea; 5 Clinical Research Coordinating Center, The Catholic Medical Center, Seoul, Republic of Korea; 6 Graduate School of Public Health, The Catholic University of Korea, Seoul, Republic of Korea; 7 College of Nursing, Catholic University of Daegu, Daegu, Republic of Korea

**Keywords:** Bone marrow stem cell, Acute myocardial infarction, Blindness, Systematic review

## Abstract

**Introduction:**

Although blinding is a methodologic safeguard to ensure obtaining comparability of groups in a clinical trial, it is very difficult to maintain blinding from the beginning to the end of a study. The aim of the study was to see how proper blinding of both participants and treatment providers from the planning phase of the study to during the study affected the study outcomes.

**Methods:**

We searched Medline, EMBASE, and Cochrane databases from inception to November 2011. The studies included in this review were randomized controlled trials, with acute myocardial infarction (AMI) patients who received percutaneous coronary intervention (PCI), intracoronary (IC) infusion of autologous bone marrow stem cells (BMSCs), unselected BMSCs, 10^8^ or more cell dose, and up to 6-month follow-up periods.

**Results:**

The initial search identified 881 references, of which 17 references were eligible for inclusion. Six of 17 trials isolated cells directly from bone marrow by aspiration in the control group as well as in the BMSC group. Nine of 17 trials underwent both cardiac catheterization and an identical injection procedure on the control group as well as the BMSC group.

Compared with the control group, BMSC transplantation improved left ventricular ejection fraction (LVEF) by 2.51 (95% CI, 1.20 to 3.83; *P* = 0.0002; *I*^*2*^ = 75%) at 6 months. In the present results, the studies that did not perform bone marrow aspiration in the control group showed significant improvement in LVEF by 3.81% (95% CI, 2.44 to 5.17), whereas no significant treatment effect was found in the studies in which the control group underwent bone marrow aspiration, as indicated the LVEF change of −1.29% (95% CI, 4.15 to 1.58). The trials that did not conduct catheterization on control subjects showed significant LVEF changes (4.45%; 95% CI, 2.48 to 6.43); however, those with cardiac catheterization as a sham procedure on the control group did not show significant changes in LVEF at 6 months (0.92%; 95% CI, -0.61 to 2.44).

**Conclusions:**

Unblinding might be overestimating the treatment effect. These findings suggest that randomized controlled trials testing the efficacy of BMSC therapy should be appropriately designed and rigorously applied to avoid bias.

## Introduction

Bone marrow stem cell (BMSC) therapy has been suggested to be safe for acute myocardial infarction (AMI) patients. However, the efficacy of this approach for cardiac repair remains controversial. A recent meta-analysis showed that intracoronary mononuclear BMSC transfer after AMI improved left ventricular ejection fraction (LVEF) significantly (2.87%, 95% CI: 2.00-3.73) after a 1-year follow-up [1]. However, these benefits were mixed, and a very high degree of heterogeneity among studies has been found. One of the reasons for this heterogeneity was the differences among the study designs. There has been debate regarding whether maintaining strict blindness in stem cell clinical research study designs is appropriate, because too-rigorous study design entails ethical issues. Some studies have maintained patient blindness in treatment allocation through the bone marrow aspiration of the control group with intracoronary infusion of BMSCs or cell-free solution [2-7]. In addition, human serum or plasma has been used to ensure that the color and consistency of the solution should be matched with that of the BMSC product. Some studies used cardiac catheterization as a sham procedure in control group patients to maintain the blindness of heath care providers. In these studies, a catheter was positioned in the stent segment, and the BMSC or a placebo was infused through the catheter. With this method, the placebo and active treatment could not be visually distinguished. In addition, the healthcare provider who conducted the surgery was not provided with any information on whether the patients belonged to the treatment or the control group, ensuring rigorous double blinding.

The randomized controlled trial is regarded as the strongest study design for assessing the benefit and harm of healthcare interventions. However, randomization in itself does not guarantee that trial results are valid. Methodologic issues affecting the validity of randomized controlled trials can occur both before and after assignment. Pildal *et al*. (2008) underlined the importance of allocation concealment to assess the intervention effects in randomized controlled trials appropriately. Two thirds of conclusions favoring an intervention would lose support if trials with unclear or inadequate allocation concealment were excluded from the meta-analysis [8]. Blinding in a randomized controlled trial is the process of masking treatment-allocation information. Unblinded participants may be affected by biases in reporting their symptoms, willingness to continue in the study, use of other effective intervention methods, and placebo effects. Unblinded healthcare providers may also cause biases through differential uses of other effective interventions, advice to patients as to whether to continue in the trial, and influencing patient reporting of outcomes [9,10].

Although blinding is difficult to include in surgical trials, it is important to consider the blinding status of the groups when evaluating the methodology of any surgical randomized controlled trial. It is unsurprising that many studies have found that such trials can overestimate the treatment effect by a substantial degree when compared with adequately concealed randomized controlled trials [11-13]. However, it is very difficult to maintain blinding from the beginning to the end of a study while using a consistent method. Once the blinding is broken, the study results may be overestimated or underestimated because of either study-participant or treatment-provider bias. An analysis of previously published studies on therapeutic trials of treatments for AMI showed that unblinded-randomized studies yield estimates of effect about 16% larger compared with blinded-randomized trials [11]. The randomized controlled trial continues to be the best method for obtaining group comparability in a clinical trial.

We conducted a systematic review and meta-analysis to investigate the impact of blinding on outcomes after intracoronary BMSCs in patients with AMI. The aim of this meta-analysis was to examine how proper blinding of both participants and treatment providers from the planning phase of the study throughout the duration of the study affected the intervention effects and study outcomes.

## Methods

### Search strategy and study selection

We searched the Medline, EMBASE, and Cochrane databases from inception to November 2011 for studies of BMSC transplantation in patients with AMI. The included studies met the following criteria: (1) randomized controlled trials, (2) AMI patients who received percutaneous coronary intervention (PCI), (3) intracoronary infusion of autologous BMSCs, (4) a cell type of unselected bone marrow stem cells, (5) a cell dose higher than 10^8^, and (6) studies that had a ≤6-month follow-up. Exclusion criteria were (1) intracoronary infusion of autologous BMSCs within 24 hours after primary PCI, (2) patients with chronic myocardial infarction (CMI), which continued at least more than 1 month after AMI, (3) AMI patients who received coronary artery bypass graft (CABG), and (4) studies published in languages other than English. We restricted the included studies by patient characteristics (AMI), cell type (unselected BMSCs), cell dose (≥10^8^), injection time (>24 hours after PCI), and follow-up period (up to 6 months) to eliminate heterogeneity due to these factors and to examine the pure effects of rigorous study design in randomized controlled trials. Statistical heterogeneity has been observed and explored previously, and it has been suggested that factors such as the timing of stem-cell infusion, the cell dose, the route of delivery, the study design, and the cell type, among others, are likely to contribute to statistical heterogeneity [14-16].

### Data extraction

Two investigators independently screened all titles and abstracts to identify studies that met the inclusion criteria and extracted relevant data, with divergences resolved by consensus. The details extracted were the study and patient-population numbers and characteristics, the type of cell dose, the route of delivery, the time of injection, the nature of the intervention and the comparator, and the follow-up period. The outcome measures included changes in left ventricular ejection fraction (LVEF), left ventricular end-systolic volume (LVESV), and left ventricular end-diastolic volume (LVEDV) from baseline to 6-month follow-up, with outcome assessment by echocardiography, magnetic resonance imaging (MRI), single-photon emission computed tomography (SPECT), or left ventricular angiography. When multiple imaging modalities were used, MRI data were preferentially included in the analysis. Clinical trials with multiple publications and sequential follow-up durations or different outcomes were considered to be one study.

### Quality assessment

Two authors independently assessed the risk of bias for each included study by using criteria based on the Cochrane Handbook for Systematic Reviews of Interventions, the principal components of which are sequence generation, allocation concealment, blinding, incomplete outcome data, and selective reporting bias [17]. Disagreements were resolved by discussion between the two authors. Assessment of methodologic quality in terms of blindness was assessed by three factors: bone marrow aspiration, cardiac catheterization, and serum or plasma infusion in both the treatment and control groups.

### Statistical analyses

Outcome data were analyzed by using Review Manager 5.1, and presented as weighted mean differences and 95% confidence intervals. Data were pooled by use of the DerSimonian-Laird random-effects model because of the high degree of heterogeneity [18]. Heterogeneity was analyzed with the *I*^2^ statistic, and heterogeneity was defined as low (25% to 50%), moderate (50% to 75%), or high (>75%). For studies that did not report the actual change from baseline to 6-month follow-up, the change in SD was calculated with a standardized formula used to calculate changes in mean and standard deviation. The extent to which the rigorous study-design effects of maintaining blindness in both patients and healthcare providers was associated with BMSC treatment effects was examined by the univariate and multivariate meta-regression model by using the PROC MIXED procedure of SAS version 9.2 (SAS Institute Inc, Cary, NC, USA).

## Results

### Search results

**Figure 1:**
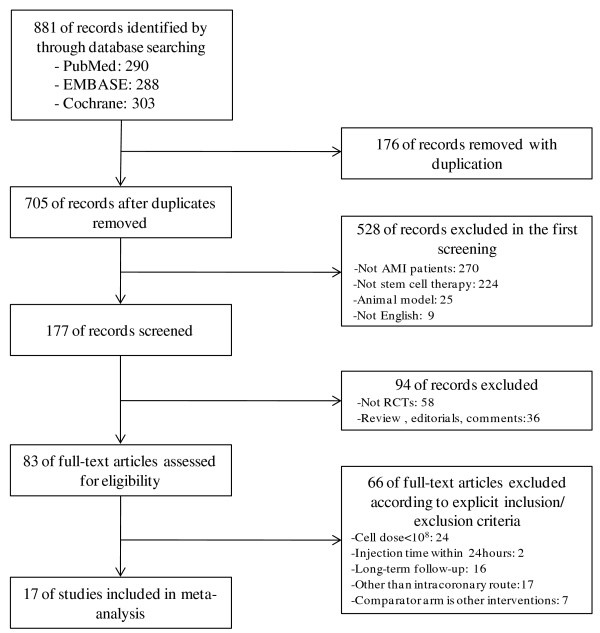
Flow diagram of studies included in this review.

The initial search identified 881 references; 705 references remained after duplicates were removed. Of these, 622 were nonrandomized studies, editorials, or reviews, and 83 of these were examined in more detail. Seventeen of the 83 references were eligible for inclusion [2-7,19-29] (Figure 1).

### Characteristics of the included studies

All trials compared unselected bone marrow stem cell treatment with a control group with patients with AMI and used PCI as a primary intervention. The presence of AMI was diagnosed by cardiologists at each study site by using such cardiac measurement tools as MRI, echocardiography, and angiography. Of the 1,072 individuals, 607 were randomized into BMSC groups and 465 into control groups aged between 18 and 80 years. All patients were recruited consecutively during the study periods. The sample size in each trial ranged from 10 to 204 participants. The follow-up duration was 6 months in all but four trials: one was 3 months, and three were 4 months. The dosage of BMSCs ranged from 1 × 10^8^ to 26.4 × 10^9^. The time to BMSC transfer from the onset of AMI ranged from 2 to 21 days. The baseline ejection fraction ranged from 32.4% to 59.6% (mean LVEF, 44.8%). The characteristics of the included studies are summarized in Table 1.

**Table 1:** Characteristics of included studies

**Study**	**BMSC (*n*)**	**Control (*n*)**	**Cell dose**	**Injection time**	**Baseline LVEF (%)**	**Follow-up**
Cao *et al.*[19]	41	45	5 × 10^8^	7 days	38.8	6 mo
Chang *et al*. [20]	20	20	1.5 × 10^9^	3 days	52.9	6 mo
Grajeck *et al.*[21]	31	14	0.41 × 10^9^	4–5 days	55.5	6 mo
Herbots *et al.*[2]	33	34	3.04 × 10^8^	4–7 days	55.5	4 mo
Huikuri *et al.*[3]	36	36	4.02 × 10^8^	2.5 days	56.5	6 mo
Meluzin *et al.*[22]	20	20	1 × 10^8^	5–9 days	40.5	6 mo
Penicka *et al.*[23]	17	10	26.4 × 10^8^	4–11 days	39	4 mo
Piepoli *et al.*[24]	19	19	2.48 × 10^8^	4 days	38.7	6 mo
Plewka *et al.*[25])	40	20	1.44 × 10^8^	7 days	34	6 mo
Schachinger *et al.*[4]	101	103	2.36 × 10^8^	3–6 days	47.1	4 mo
Suarez de Lezo *et al*. [26]	10	10	9 × 10^8^	7 days	38	3 mo
Tendera *et al.*[27]	80	40	1.78 × 10^8^	7 days	37	6 mo
Traverse *et al.*[5]	30	10	1.5 × 10^8^	4.5 days	38	6 mo
Traverse *et al.*[6]	58	29	1.5 × 10^8^	14–21 days	47	6 mo
Wohrle *et al.*[7]	29	13	3.81 × 10^8^	5–7 days	59.6	6 mo
Wollert *et al.*[28]	30	30	24.6 × 10^8^	4.8 days	50.7	6 mo
Yao *et al.*[29]	12	12	2.0 × 10^8^	3–7 days	32.4	6 mo

*BMSC* bone marrow stem cell, *LVEF* left ventricular ejection fraction.

To examine the effect of rigorous study design in maintaining the blinding of both patients and healthcare providers, we extracted data with respect to the following three factors. First, to evaluate patient-treatment blindness, we examined whether both the treatment and the control groups underwent bone marrow aspiration. Six of 17 trials isolated cells directly from bone marrow by aspiration in both the control group and the BMSC group. The second factor was whether cardiac catheterization was used as a sham procedure when surgeons conducted the intracoronary administration. In nine of 17 trials, cardiac catheterization was used as a sham procedure in both the BMSC and control groups to maintain blindness. Third, we also looked at whether control group patients were infused with a placebo such as their own serum, plasma, or autologous erythrocytes without BMSCs to ensure that healthcare providers remained blinded to the study conditions. In nine of 17 trials, both the BMSC and control groups underwent identical injection procedures that were visually indistinguishable from the active treatment to maintain blindness (Table 2).

**Table 2:** Rigorousness of methodology

**Study**	**Bone marrow aspiration in all patients**	**Comparator arm**	**Cardiac catheterization in all patients**	**Infused serum without cell in the control group**
Cao *et al.*[19]	NO	Heparinized saline	YES	NO
Chang *et al*. [20]	NO	No placebo	NO	NO
Grajeck *et al.*[21]	NO	No cell therapy	YES	NO
Herbots *et al.*[2]	YES	NS w/5% serum	YES	YES
Huikuri *et al.*[3]	YES	Serum	YES	YES
Meluzin *et al.*[22]	NO	Cell suspension media	NO	NO
Penicka *et al.*[23]	NO	Standardized medicine	NO	NO
Piepoli *et al.*[24]	NO	Optimized treatment only	NO	NO
Plewka *et al.*[25])	NO	Standardized medicine	NO	NO
Schachinger *et al.*[4]	YES	Serum	YES	YES
Suarez de Lezo *et al.*[26]	NO	Heparinized saline	NO	NO
Tendera *et al.*[27]	NO	No cell therapy	NO	NO
Traverse *et al.*[5]	YES	Saline w/albumin	YES	YES
Traverse *et al.*[6]	YES	Saline w/albumin	YES	YES
Wohrle *et al.*[7]	YES	Erythrocyte	YES	YES
Wollert *et al.*[28]	NO	Medical treatment	NO	NO
Yao *et al.*[29]	NO	Heparinized saline	YES	NO

### Quality assessment

Study quality was assessed on the basis of randomization, allocation concealment, blinding of outcome assessment, and adequacy of follow-up. The overall quality of the included trials was good (Table 3).

**Table 3:** Methodologic quality assessment of included studies

**Study**	**Randomization**	**Allocation concealment**	**Blinding of outcome assessment**	**Attrition**
Cao *et al.*[19]	Y	Y	Y	Y
Chang *et al*. [20]	Y	U	Y	Y
Grajeck *et al.*[21]	Y	Y	Y	Y
Herbots *et al.*[2]	Y	Y	U	Y
Huikuri *et al.*[3]	Y	Y	Y	Y
Meluzin *et al.*[22]	U	U	U	Y
Penicka *et al.*[23]	U	U	U	Y
Piepoli *et al.*[24]	N	N	Y	Y
Plewka *et al.*[25])	U	U	Y	Y
Schachinger *et al.*[4]	Y	Y	Y	Y
Suarez de Lezo *et al.*[26]	Y	Y	Y	Y
Tendera *et al.*[27]	Y	Y	Y	N
Traverse *et al.*[5]	Y	Y	Y	Y
Traverse *et al.*[6]	Y	Y	Y	Y
Wohrle *et al.*[7]	Y	U	Y	Y
Wollert *et al.*[28]	Y	Y	Y	Y
Yao *et al.*[29]	Y	Y	Y	Y

*Y* low risk of bias, *U* unclear; *N* high risk of bias.

### Main findings

Compared with the control group, BMSC transplantation improved LVEF by 2.51% (95% CI, 1.20 to 3.83; *P* = 0.0002; *I*^*2*^ = 75%) at 6 months. BMSC transplantation was similarly found to reduce LVESV by 4.98 ml (95% CI, -7.65 to −2.31; *P* = 0.0003; *I*^*2*^ = 73%). A trend of reduction in LVEDV was observed of 3.46 ml (95% CI, -7.62 to 0.69; *P* = 0.1; *I*^*2*^ = 86%).

**Figure 2:**
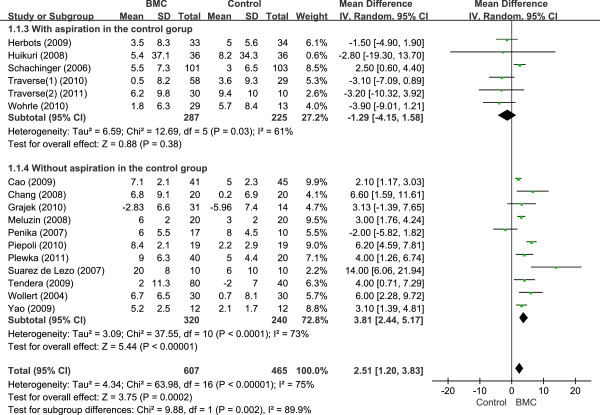
Forest plot of mean difference in LVEF with or without bone marrow aspiration.

To examine the effect of rigorous study design on maintaining blindness in both patients and healthcare providers, we conducted separate analyses with respect to the sham procedures, including bone marrow aspiration and infusion of patients’ own serum for blinding the patients and cardiac catheterization for blinding the healthcare providers in the control group. The trials were categorized into two groups according to whether the studies used these procedures in the control group. Six of 17 trials isolated cells directly from bone marrow by aspiration and infused patients with their own serum in the control group as well as the BMSC group. No significant treatment effect was seen in six studies in which the control group underwent bone marrow aspiration, as indicated by an LVEF change of −1.29% (95% CI, −4.15 to 1.58). However, the rest of the 11 studies that did not perform bone marrow aspiration in the control group showed significant improvement in LVEF by 3.81% (95% CI, 2.44 to 5.17) (Figure 2).

**Figure 3:**
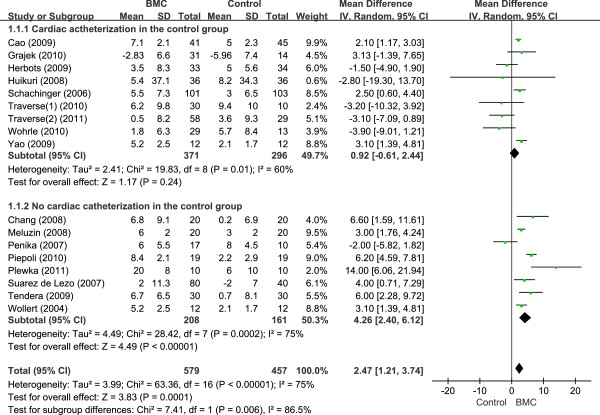
Forest plot of mean differences in LVEF with or without cardiac catheterization.

Nine of 17 trials included cardiac catheterization in both the BMSC and the control group to maintain blindness to treatment. Trials that used cardiac catheterization in the control group did not show significant changes in LVEF at 6 months (0.92%; 95% CI, -0.61 to 2.44); however, those without catheterization in control subjects showed significant LVEF changes (4.45%; 95% CI, 2.48 to 6.43) (Figure 3). Both LVESV and LVEDV reduction were also overestimated, according to the rigorousness of the study design (Table 4).

**Table 4:** Comparing the difference LVESV and LVEDV with or without bone marrow aspiration and cardiac catheterization in the control group

	**LVESV**	**LVEDV**
	**WMD (95% CI)**	**WMD (95% CI)**
BM aspiration in the control group		
Yes	−2.77 (−6.27 to 0.74)	−1.88 (−9.80 to 6.04)
No	−6.39 (−10.02 to −2.77)	−4.54 (−9.87 to 0.79)
Cardiac catheterization in the control group		
Yes	−3.72 (−6.35 to −1.09)	−2.40 (−7.75 to 2.94)
No	−7.31 (−13.45 to −1.17)	−4.97 (−11.52 to 1.57)

We conducted meta-regression analyses to explore the source of heterogeneity. The results showed that rigorous studies that maintained the blindness of both patients and healthcare providers were negatively associated with the BMSC treatment effects, even after being controlled for the effects of randomization, allocation concealment, cell dose, injection time, and baseline LVEF (Table 5).

**Table 5:** Univariate and multivariate meta-regression analyses of BMSC treatment effects

**Study characteristics**	**Univariate analysis**		**Multivariate analysis**^**c**^	
	**Mean difference (95% CI)**	***P***	**Mean difference (95% CI)**	***P***
Bone marrow aspiration^a^	−3.27 (−3.89 to −2.65)	<0.0001	−6.62 (−9.65 to −3.59)	0.0003
Catheterization^b^	−0.54 (−3.57 to 2.50)	0.688	−5.55 (−9.35 to −1.76)	0.0071
Effects of study quality				
Randomization	1.23 (−2.62 to 5.08)	0.474	−2.62 (−6.16 to 0.92)	0.135
Allocation concealment	0.43 (−3.54 to 4.39)	0.807	−0.05 (−4.69 to 4.59)	0.982
Effects of study characteristics				
Cell dose	2.23 (−0.10 to 4.55)	0.059	1.45 (−3.42 to 6.32)	0.535
Injection time	2.10 (−0.15 to 4.35)	0.065	1.46 (−3.48 to 6.39)	0.538
Baseline LVEF	1.69 (−0.45 to 3.84)	0.114	3.40 (−1.66 to 8.46)	0.172

^a^Bone marrow aspiration in the control group. ^b^Catheterization in the control group. ^c^Adjusted by randomization, allocation concealment, cell dose, injection time, and baseline LVEF.

## Discussion

The present meta-analysis included 17 RCTs in which BMSC treatment was compared with a control group. The results demonstrated that, at 6 months, BMSC treatment leads to a 2.51% improvement in LVEF, a 4.98-ml reduction in LVESV, and a 3.46-ml reduction in LVEDV. Similar results were obtained in recent meta-analyses [30,31]. Taken together, BMSC treatment improved LVEF outcome at 6 months.

We stress some important issues concerning rigorous study design in randomized controlled trials. All of the RCTs in the present analysis conducted random allocation to treatment and control groups. However, some researchers made an effort to maintain blindness to patients as well as healthcare providers in regard to group allocation, whereas others did not. Clinical trials of stem cell therapy should be strictly designed, as it is very difficult to maintain blinding throughout the entire research period, even when research is designed with double-blind controlled trials [8]. We considered two procedures that are performed to maintain the blindness of treatment allocation to both patients and healthcare providers. First, bone marrow is harvested in all patients, including the control group, under full anesthesia just before surgery to ensure that the characteristics of the BMSC suspension do not differ significantly between the two groups [2-7]. Second, patients are returned to cardiac catheterization after bone marrow aspiration to ensure identical injection procedures in all patients. It is assumed that using autologous erythrocytes or patients’ own serum or plasma in the placebo preparation ensures double blindness. In the present results, no significant treatment effect was found in studies in which the control group underwent bone marrow aspiration, as indicated by the LVEF change of −1.29% (95% CI, −4.15 to 1.58), whereas studies that did not perform bone marrow aspiration in the control group showed significant improvement in LVEF by 3.81% (95% CI, 2.44 to 5.17). The intervention effect might therefore be overestimated because of the study design. Several trials used placebo preparations without cells in which the content of the syringes could be easily distinguished between the active treatment and the placebo. It is important that BMSCs were harvested in all patients, including the control group, under full anesthesia just before surgery, to guarantee that the characteristics of the BMSC suspension did not differ significantly between groups.

Nine of 17 trials implemented identical cardiac catheterization injection procedures after bone marrow aspiration in both the BMSC and control groups to maintain study blindness [2-7,19,21]. The surgeon was unaware whether cells or only saline was being injected. Trials that conducted cardiac catheterization as a sham procedure in the control group did not show significant changes in LVEF at 6 months (0.92%; 95% CI, -0.61 to 2.44); however, those without catheterization of control subjects showed significant LVEF changes (4.45%; 95% CI, 2.48 to 6.43).

One of the major differences of this study from other preexisting studies is that only conditions known to be effective in bias-minimized RCT studies were selected to evaluate how the rigorousness of method affected treatment effects. All included studies included PCI before the infusion of BMSC treatment or placebo. We included only trials in which the cell dose was higher than 10^8^. Previous trials determined that the use of 10^8^ or more injected cells shows improved outcomes in BMSC-treated patients [15,30]. The mean change in LVEF was statistically significant in favor of administering BMSCs in studies using higher doses of BMSCs. These results suggest that significant effects on LVEF may be achieved only when the infusing doses are higher than 10^8^ BMSCs. The route of BMSC delivery was intracoronary artery infusion by using a balloon in all included studies, as the present meta-analysis included only AMI patients. All included trials reported up to 6 months of short-term follow-up data to demonstrate the effects of rigorous study design.

We excluded whether the BMSCs were administered within 24 hours after primary PCI, because it was previously shown that the timing of cell transfer does not have a BMSC treatment effect in AMI patients [31,32]. A larger randomized study suggested that BMSCs should ideally be administrated more than 4 days after STEMI to obtain the best benefit from this therapy [4]. In a subgroup analysis, the REPAIR-AMI Trial found that the most favorable effects on LV function were observed with BMSC delivery on days 5 to 7 after MI [4].

We chose the studies to be included based on study design, route of delivery, cell type, cell dose, timing of injection, and follow-up duration to reduce heterogeneity and to estimate the effect of rigorous study design in randomized controlled trials. Heterogeneity was reduced from between 85% and 98% to 75% in the present study. When only rigorously designed studies were included in the analysis, heterogeneity was further reduced to 60%. Meta-regression analyses, exploring the source of heterogeneity, indicated that the treatment effects seen were associated with rigorous study designs. These findings were similar to those of the subgroup analyses. Design rigorousness seemed to explain the heterogeneity in the studies; however, a considerable degree of heterogeneity was still observed among the included trials. This might be due to differences in patient severity at baseline, the timing of infusions, or the duration of follow-up between studies.

## Conclusions

These findings suggest that randomized controlled trials testing the effectiveness of BMSC therapy should be appropriately designed and rigorously applied to avoid bias. Blinding might overestimate the treatment effect. If properly designed, conducted, and interpreted, study results are likely to make a substantial impact on the outcomes of patients.

## Abbreviations

AMI: Acute myocardial infarction; BMSC: Bone marrow stem cell; IC: Intracoronary; LVEDV: Left ventricular end-diastolic volume; LVEF: Left ventricular ejection fraction; LVESV: Left ventricular end-systolic volume; MRI: Magnetic resonance imaging; PCI: Percutaneous coronary intervention; SPECT: Single-photon emission computed tomography.

## Competing interests

The authors declare that they have no competing interests.

## Authors’ contributions

HJ participated in eligibility screening, data extraction, preparation of the protocol, analysis and interpretation of data, and writing the manuscript; HWY, scientific concept expert, eligible screening, data extraction, quality assessment data analysis, analysis and interpretation of data, preparation of the protocol, and scientific guidance, and critical review of the manuscript; YC, design and implementation of search strategies, initial eligibility screening, data verification, and critical review of the manuscript; HJP, clinical content expert, comments on protocol, and critical review of manuscript; SJ, design and implementation of search strategies and data search and validation; H-bK, design and implementation of search strategies and analysis and interpretation data; WH, initial eligibility screening, data verification, and quality assessment of data; and HK, design and implementation of search strategies, initial eligibility screening, and data verification. All authors read and approved the final manuscript.
